# Posterior leukoencephalopathy following repair of an ileocecal anastomosis breakdown: a case report and review of the literature

**DOI:** 10.1186/1752-1947-5-20

**Published:** 2011-01-19

**Authors:** Pascal O Zinn, Rivka R Colen, Ekkehard M Kasper, Clark C Chen

**Affiliations:** 1 Department of Neurosurgery, Beth Israel Deaconess Medical Center, Harvard Medical School, Boston MA 02115, USA; 2 Department of Radiation Oncology, Dana-Farber Cancer Institute, Harvard Medical School, Boston MA 02115, USA; 3 Department of Radiology, Brigham and Women's Hospital, Harvard Medical School, Boston MA 02115, USA

## Abstract

**Introduction:**

Posterior reversible leukoencephalopathy syndrome refers to a constellation of neurologic symptoms related to temporary white matter changes. The disease typically presents in the context of an abrupt and drastic elevation in blood pressure (>180/110 mmHg). We report an unusual case of posterior reversible leukoencephalopathy syndrome in a post-operative setting, with a blood pressure parameter generally tolerated by most patients.

**Case presentation:**

We report the case of a 22-year-old Caucasian man who suffered acute onset visual acuity loss four days after an ileocecal anastomosis. A head magnetic resonance imaging scan revealed findings typically associated with posterior reversible leukoencephalopathy syndrome. His symptoms developed in the context of blood pressure parameters that are typically well tolerated in a post-operative setting (150-160/80-90 mmHg). He did not have a history of renal failure or immunosuppression. His symptoms resolved with aggressive blood pressure management.

**Conclusions:**

Posterior reversible leukoencephalopathy syndrome can occur in a post-operative setting with blood pressure parameters typically well-tolerated in most post-surgical patients. Timely diagnosis and treatment will minimize the risk of permanent neurologic injury.

## Introduction

Posterior reversible leukoencephalopathy syndrome (PRES) refers to a constellation of neurologic symptoms related to temporary white matter changes [1]. Clinically, it is characterized by a constellation of symptoms including the acute onset of headache, nausea, vomiting, visual changes, altered mental status, seizures, and focal neurologic deficits [2]. The most characteristic radiographic feature involves edema of the subcortical white matter in the posterior cerebral parenchyma [1,3]. This syndrome is typically associated with acute onset severe hypertension (>180/110 mmHg) or with the use of immunosuppressive medications [1,4].

While extreme cases of PRES may progress to infarction and hemorrhage despite appropriate blood pressure control [3], the neurologic deficits associated with PRES typically resolve with timely blood pressure control or discontinuation of immunosuppression. A failure to recognize this syndrome and initiate the correct treatment can increase the likelihood of permanent neurologic injuries [5]. The importance of recognizing this syndrome is underscored by the fact that the symptoms of PRES often mimic those of bilateral posterior cerebral artery infarcts [6]. The treatment of the former typically involves anti-hypertensive medication or the withdrawal of offending agents, while the latter generally requires the induction of hypertension.

We report a case of PRES in a man with mild hypertension (150-160/80-90 mmHg) following the repair of an ileocecal anastomosis breakdown. This case is of interest because PRES occurred in the context of blood pressure parameters that are generally well tolerated in post-operative patients, and our patient harbored no other risk factors for PRES. Through our case report and a review of the literature, we hope to heighten an awareness of this syndrome, particularly in a post-operative setting.

## Case presentation

**Figure 1:**
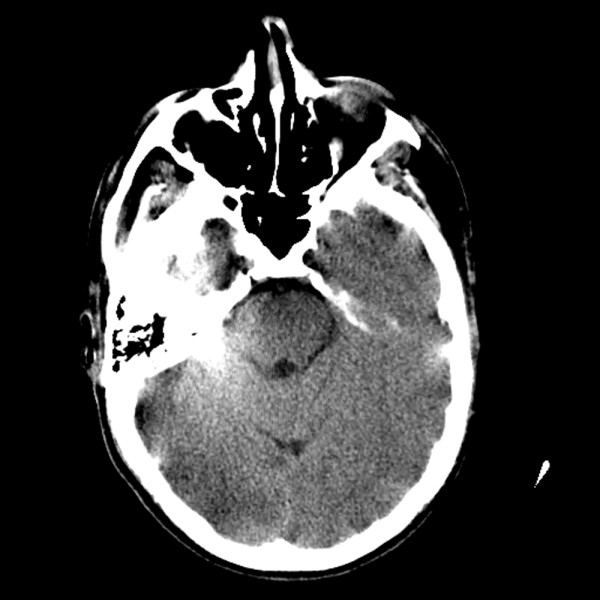
**A head CT without contrast demonstrating subtle, ill-defined regions of low attenuation in the bilateral temporo-occipital lobes**.

**Figure 2:**
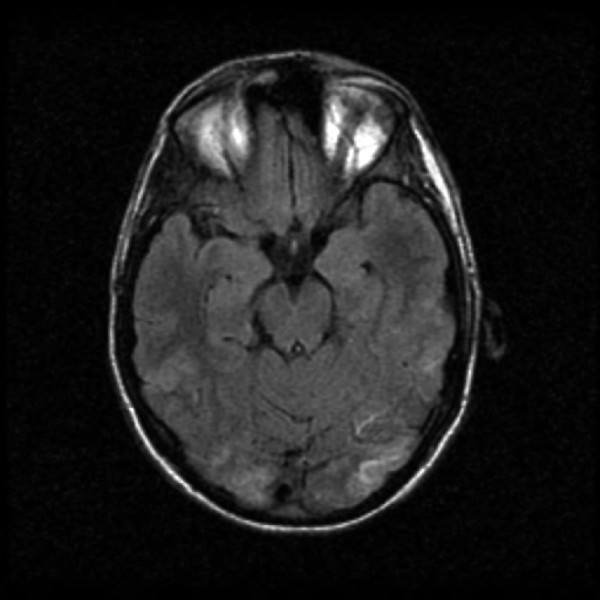
**A T2-weighted MRI image of his head shows bilateral, slightly asymmetric, multifocal T2 hyperintensities in the posterior temporo-occipital subcortical white matter**. There were no DWI (image not shown) signal abnormalities to suggest ischemia. These findings are most consistent with vasogenic edema. Post-gadolinium images (image not shown) demonstrated no abnormal enhancement.

We report the case of a 22-year-old Caucasian man with a 10-year history of Crohn's disease. He had recently undergone a small bowel resection and ileocecal anastomosis. On his fourth post-operative day, he experienced a breakdown of his anastomosis and underwent surgical repair. He had no other medical co-morbidities and was not being treated with immunosuppressive agents. On post-operative day three, he developed symptoms of lightheadedness, headache, and blurred vision that progressed to visual loss. An emergent ophthalmology evaluation revealed poor visual acuity (he was only able to count fingers at 3 inches bilaterally) but no fundoscopic or ocular abnormality. His neurologic examination was otherwise unremarkable. Shortly thereafter, he developed a tonic-clonic seizure that abated spontaneously after approximately one minute. His blood pressure throughout the episode was 150-160/80-90 mmHg, up from a baseline of 100-130/70-80 mmHg. His serial electrolytes (including calcium), arterial blood gas, cardiac enzymes, urine analysis, and electrocardiogram were within normal limits. A head computed tomography (CT) scan demonstrated subtle, ill-defined regions of low attenuation involving his bilateral posterior temporo-occipital lobes (Figure 1). A head magnetic resonance imaging (MRI) scan confirmed patchy T2 hyperintensities in his bilateral temporo-occipital subcortical white matter, consistent with vasogenic edema (Figure 2). There were no diffusion weighted imaging (DWI) or apparent diffusion coefficient (ADC) signal abnormalities to suggest an acute infarction. Given our clinical and classical radiological findings, he was diagnosed with PRES.

His symptoms and the imaging findings resolved with an aggressive blood pressure control. A subsequent ocular examination showed him to have a visual acuity of 20/40 OD and 20/30 OS. A follow-up MRI was performed three days after his symptoms had resolved. This MRI showed the complete resolution of his bilateral temporo-occipital subcortical white matter T2 signal abnormalities. He continued to do well at his two-year follow-up examination.

## Discussion

We present the case of a 22-year-old man who developed PRES following the repair of an ileocecal anastomosis breakdown with blood pressure parameters generally tolerated by most post-surgical patients. To date, four cases of PRES have been reported in an immediate post-operative period [2,3,7,8]. All the reported cases of post-operative PRES occurred in the context of severe hypertension, where blood pressure was elevated beyond 180/110 mmHg. Moriarity *et al. *described the case of a normotensive 19-year-old man who suffered two generalized tonic-clonic seizures while emerging from anesthesia. A review of his intra-operative anesthesia records revealed hypertensive episodes with blood pressures up to 200/130 mmHg [2]. Ay *et al. *reported the case of a 66-year-old woman who underwent an oophorectomy for ovarian cancer and subsequently developed cortical blindness on her fourth post-operative day in the context of blood pressures ranging from 180-190/90-100 mmHg. Her baseline blood pressure was 160-170/60-70 mmHg [3]. A third report presented the case of a normotensive 54-year-old woman who underwent a colectomy for ischemic colitis. On the 35th post-operative day, her blood pressure elevated to 200/85 mmHg and she suffered a tonic-clonic seizure [3]. In the fourth report, Triquenot-Bagan *et al. *reported the case of a 55-year-old man who was operated on for an abdominal aortic aneurysm and developed a severe diffuse headache with vomiting eight days after the surgical intervention. A neurological examination was positive for cortical blindness, and his blood pressure was 180/110 mmHg [7]. It is worthy of note that one of the four reported cases of PRES was initially misdiagnosed as bilateral posterior cerebral artery infarctions [3]. Timely MRI and CT imaging as well as an awareness of the diagnosis of PRES are key in avoiding a misdiagnosis.

Our case report is unusual in that PRES developed in the context of blood pressure parameters that are generally well tolerated in days two to four of the post-operative period (150-160/80-90 mmHg). Such transient, benign hypertension is frequently seen in a post-operative setting and generally resolves spontaneously. Recognition of the possibility that PRES can occur in this setting is critical for a timely diagnosis and treatment.

Given the complexity of the post-operative state and the limitations of a case report, it is difficult to identify the pathogenesis in our case report. The pathophysiologic mechanism of PRES is unknown, although it is thought that PRES results from dysfunction of the cerebrovascular auto-regulatory mechanism secondary to hypertension or pharmacologic agents. It is thought that cerebral vasculature constricts in response to hypertension to prevent cerebral over-perfusion. This vasoconstriction is mediated by an increased sympathetic tone. Dysfunction in this process predisposes patients to cerebral over-perfusion and consequently, PRES. It is hypothesized that the vulnerability of the posterior cerebral parenchyma is related to the paucity of sympathetic innervation in the posterior cerebral vasculature [3]. It is conceivable that low-grade sepsis, secondary to the bowel anastomosis breakdown, might have contributed to the etiology in our case report.

## Conclusions

Through our case report and a review of the literature, we wish to highlight the fact that PRES can occur in a post-operative setting with blood pressure parameters usually well tolerated in most post-surgical patients. An awareness of this observation is crucial for a timely diagnosis and treatment, in order to minimize the risk of permanent neurologic deficits.

## Consent

Written informed consent was obtained from the patient for publication of this case report and any accompanying images. A copy of the written consent is available for review by the Editor-in-Chief of this journal.

## Competing interests

The authors declare that they have no competing or financial interests.

This study was conducted according to HIPAA/IRB guidelines by the Harvard Medical School, MGH, and Beth Israel Deaconess Medical Center.

## Authors' contributions

POZ, RRC, EMK, CCC wrote the manuscript. All authors read and approved the final manuscript.
